# A *COLQ* Missense Mutation in Sphynx and Devon Rex Cats with Congenital Myasthenic Syndrome

**DOI:** 10.1371/journal.pone.0137019

**Published:** 2015-09-01

**Authors:** Marie Abitbol, Christophe Hitte, Philippe Bossé, Nicolas Blanchard-Gutton, Anne Thomas, Lionel Martignat, Stéphane Blot, Laurent Tiret

**Affiliations:** 1 Inserm, IMRB U955-E10, 94000, Créteil, France; 2 Université Paris Est, Ecole nationale vétérinaire d'Alfort, 94700, Maisons-Alfort, & Faculté de médecine, 94000, Créteil, France; 3 Etablissement Français du Sang, 94017, Créteil, France; 4 APHP, Hôpitaux Universitaires Henri Mondor, DHU Pepsy & Centre de référence des maladies neuromusculaires GNMH, 94000 Créteil, France; 5 Institut de Génétique et Développement de Rennes IGDR, UMR6290 CNRS—Université de Rennes 1, Rennes, France; 6 Antagene, Animal Genetics Laboratory, La Tour de Salvagny, France; 7 ONIRIS, UP Sécurité Sanitaire en Biotechnologies de la Reproduction, Nantes, France; Institut de Myologie, FRANCE

## Abstract

An autosomal recessive neuromuscular disorder characterized by skeletal muscle weakness, fatigability and variable electromyographic or muscular histopathological features has been described in the two related Sphynx and Devon Rex cat breeds (*Felis catus*). Collection of data from two affected Sphynx cats and their relatives pointed out a single disease candidate region on feline chromosome C2, identified following a genome-wide SNP-based homozygosity mapping strategy. In that region, we further identified *COLQ* (*collagen-like tail subunit of asymmetric acetylcholinesterase*) as a good candidate gene, since *COLQ* mutations were identified in affected humans and dogs with endplate acetylcholinesterase deficiency leading to a synaptic form of congenital myasthenic syndrome (CMS). A homozygous c.1190G>A missense variant located in exon 15 of *COLQ*, leading to a C397Y substitution, was identified in the two affected cats. C397 is a highly-conserved residue from the C-terminal domain of the protein; its mutation was previously shown to produce CMS in humans, and here we confirmed in an affected Sphynx cat that it induces a loss of acetylcholinesterase clustering at the neuromuscular junction. Segregation of the c.1190G>A variant was 100% consistent with the autosomal recessive mode of inheritance of the disorder in our cat pedigree; in addition, an affected, unrelated Devon Rex cat recruited thereafter was also homozygous for the variant. Genotyping of a panel of 333 cats from 14 breeds failed to identify a single carrier in non-Sphynx and non-Devon Rex cats. Finally, the percentage of healthy carriers in a European subpanel of 81 genotyped Sphynx cats was estimated to be low (3.7%) and 14 control Devon Rex cats were genotyped as wild-type individuals. Altogether, these results strongly support that the neuromuscular disorder reported in Sphynx and Devon Rex breeds is a CMS caused by a unique c.1190G>A missense mutation, presumably transmitted through a founder effect, which strictly and slightly disseminated in these two breeds. The presently available DNA test will help owners avoid matings at risk.

## Introduction

Congenital myasthenic syndromes (CMSs) are genetically heterogeneous inherited diseases characterized by a defect in signal transmission at the neuromuscular junction (NMJ). In humans, CMSs are predominantly autosomal recessive morbidity traits and altogether, were initially classified according to the location of the causative protein as presynaptic, synaptic or postsynaptic forms. In the recent years, mutations in 20 genes involved in neuromuscular signal transmission and neuromuscular endplate integrity were characterized, leading to the recognition of new unclassified forms of CMSs [1]. Spontaneous animal models for this group of diseases are rare, but were described in dogs [2–7] and cows [8]; to date, molecular aetiology was deciphered only in Old Danish Pointing Dogs [6], Labrador Retrievers [7] and Brahman cows [8].

Clinically, skeletal muscle weakness and fatigue represent common, non-specific features of CMSs in humans and animals, which diagnosis is challenging in most cases because of a highly variable clinical expression and the absence of specific histological anomalies or routine electromyographic responses. To date, only the molecular identification of a causing-mutation helps confirm the CMS form [1, 9]. Synaptic forms of CMSs are endplate acetylcholinesterase deficiencies mainly due to mutations in the *COLQ (collagen-like tail subunit of asymmetric acetylcholinesterase)* gene or more rarely, in the *LAMB2 (laminin beta 2)* gene (reviewed in [1]). At the neuromuscular junction, acetylcholinesterase (AChE) terminates signal transmission by hydrolysing the neurotransmitter acetylcholine. COLQ is a key protein of the NMJ, crucial for anchoring AChE to the basal lamina through its three domains. The COLQ NH_2_-terminal proline-rich domain assembles the C-terminal domain of AChE; the central collagenic domain interacts with the transmembrane dystroglycan complex through the proteoglycan perlecan; the C-terminal domain interacts with the muscle-specific receptor tyrosine kinase (MuSK) and proteins of the basal lamina, yet to be identified [10–12].

In the domestic cat (*Felis catus*), few inherited neuromuscular disorders have been reported (omia.angis.org.au). An autosomal recessive muscular disorder initially named “spasticity” was reported to segregate in the two Sphynx and Devon Rex feline breeds [13, 14]. This disease first reported in the Devon Rex breed in 1974 was further characterized by Malik and collaborators in 1993 who concluded to a primary muscular dystrophy [15]. Even though primarily described in the Devon Rex breed, it may also affect the Sphynx breed [13]. Indeed, Sphynx and Devon Rex breeds are genetically very close, because of the repeated use of Devon Rex cats in Sphynx breeding programs [16]. In the first five months of their life, affected cats from the two breeds display a general muscle weakness with a more pronounced functional deficiency of limb-girdle and axial muscles resulting in severe locomotor difficulties, fatigability, dorsal protrusion of the scapulae and passive ventroflexion of the head and neck [13, 15]. Therefore, the Sphynx and Devon Rex muscular disorder shares features with limb-girdle muscular dystrophies affecting skeletal muscles of the pelvic and shoulder girdles in humans [17] and with dystroglycanopathies that result from a deficiency in a dystroglycan protein [13]. To date, the underlying mutation for this feline disease remained unknown.

Here we report the identification of a missense recessive mutation in *COLQ* leading to impaired clustering of AchE, suggesting that the Sphynx and Devon Rex neuromuscular disease is a congenital myasthenic syndrome, the first one described in cats.

## Material and Methods

### Animals

The two female Sphynx littermates included in this study were breeder's cats spontaneously presented at the Neurological clinics located at the Alfort School of Veterinary Medicine in Maisons-Alfort, France. They were clinically evaluated prior to electrophysiological examination, blood and muscle biopsies collection; the latter were performed under general anaesthesia. Routine blood cells count and serum biochemical parameters quantification, including creatine kinase activity, were performed on blood samples.

A total of 333 cats from 14 breeds were included in the genetic study. They were sampled in Europe, from January 2007 to March 2015 and included individuals from the following breeds: Sphynx (n = 107, including the affected sib-pair, 24 healthy cats from the family and 81 control cats), Devon Rex (n = 15, including one affected cat and 14 controls), Persian (n = 20), Chartreux (n = 16), Birman (n = 27), British shorthair and longhair (n = 20), Norwegian Forest (n = 22), Siamese and Oriental shorthair and longhair (n = 22), Maine Coon (n = 21), Abyssinian (n = 7), Ragdoll (n = 10), Bengal (n = 15), Domestic shorthair and longhair (n = 27) and Russian Blue (n = 4). All cats were included following their owners’ consent. For the unaffected Sphynx cats from the affected family and for the affected Devon Rex cat, non-invasive cheek swabs were sent back directly by owners or collected by a veterinarian. For additional Sphynx and Devon Rex cats and other breeds, buccal swabs were collected for genetic studies through a feline DNA banking initiative. Non-invasive buccal swabs were sent back directly by owners or collected by a veterinarian. Pedigrees were collected from the owners. Genealogical data were drawn using GenoPro (www.genopro.com).

### Ethics Statement

All animals were client-owned cats on which no harmful invasive procedures were performed, so there was no animal experimentation according to the legal definition in Europe (Subject 5f of Article1, Chapter I of the Directive 2010/63/UE of the European Parliament and of the Council).

Invasive procedures in the two Sphynx female cats were performed for medical reasons, at the request and with the consent of their owners who required a detailed diagnosis of the disease affecting their pets. Blood and biopsies were obtained as part of routine clinical procedures for diagnostic purposes. The DVM who performed these procedures (SB) is a board-certified veterinarian, accredited by the Veterinary Division of French Ministry of Agriculture and member of the European College of Veterinary Neurology (ECVN).

DNA of control cats were obtained non-invasively (cheek swabs) for zootechnical purposes, at the request of their owners who consented that their cats' DNA could be used in research projects aiming at improving animal health and welfare.

### Electrophysiology

Electrodiagnostics, including electromyography (EMG) and measurement of motor nerve conduction velocity (MNCV), were performed on the two littermates by a board-certified neurologist (SB) as previously described [18]. Procedures were performed under general anaesthesia, with an EMG unit (Nicolet Viking Select, Viasys Healthcare, Madison, USA). The anaesthetic protocol involved the placement of a peripheral IV catheter, induction of anaesthesia with propofol (4 mg/kg [1.8 mg/lb], IV, to effect) and maintenance with isoflurane vaporized in oxygen. The EMG was performed with a disposable, concentric 0.45-mm needle with a 0.068-mm^2^ sampling area, with the cat in a lateral recumbent position. Both thoracic and pelvic limbs were examined. Three needle passes were performed in three areas for all of the examined muscles. MNCV of the radial, ulnar, peroneal and tibial nerves were performed with polytetrafluoroethylene-coated, stainless steel monopolar needles with 3-mm bare tips for stimulations and recordings. Supramaximal stimuli of 0.1 milliseconds’ duration were delivered at a rate of 1 Hz. Repetitive nerve stimulation was not performed.

### Histology, Histochemistry and Epifluorescence

Biopsies from the two littermates (biceps femoris, triceps brachii and dorsal cervical: splenius capitis) were collected under general anaesthesia. Biopsies were immediately frozen in isopentane cooled in liquid nitrogen (-135°C), then stored at -80°C until processed. Transverse cryosections (6 μm thick) were stained using standard protocols [19], including haematoxylin-eosin, modified Gomori trichrome, oil red O, reduced nicotinamide adenine dinucleotide deshydrogenase-tetrazolium reductase and myosin adenosine triphosphatase isoforms (ATPase) of routine specific activity at pH 9.4. Fluorescent localization of acetylcholine receptors (AchR) at motor end-plate was performed by incubating sections with Alexa Fluor 488 α-bungarotoxin (1:1000, Life Technologies) for 30 minutes. Histochemical localization of acetylcholine esterase (AchE) at motor end-plate was performed with the esterase reaction (Karnowsky and Roots reaction); 5 mg of acetylthiocholine iodide were dissolved in 6.5 ml of 0.1 M sodium acetate buffer (pH 5.5). To this solution, 0.5 ml of 0.2M sodium citrate, 1 ml of 30 mM cupric sulfate, 1 ml of H_2_O, and 1 ml of 5 mM potassium ferricyanide were added and mixed. Three hundred microliters of this solution were put on sections for 30 minutes.

### DNA extraction

DNA was extracted from whole blood and buccal swabs according to the manufacturers' protocols, using either a Maxwell 16 Instrument (Promega Corporation, Madison, USA), or the NucleoSpin 96 Tissue DNA Kit (Macherey-Nagel EURL, Hoerdt, France).

### SNP genotyping

Seven Sphynx cats from the proband’s family (the proband, her affected brother, her healthy female littermate, their two parents and the two paternal grandparents) were genotyped using the Illumina Infinium iSelect 63k Cat DNA SNP genotyping array. For each cat, 1 μg of genomic DNA was send to the Labogena laboratory (www.labogena.fr). Arrays were processed according to the manufacturer’s protocol. SNP genomic positions were inferred according to the updated felCat5 SNP manifest for the Illumina Feline 63k SNP array [20].

### *COLQ* sequencing and genotyping

Reference genomic sequences were collected from Ensembl [www.ensembl.org; feline *COLQ* gene, (ENSFCAG00000011304)] or by aligning the human exon 1 for *COLQ* (ENST00000383788) in the trace archive databases for *Felis catus* (WGS; http://blast.ncbi.nlm.nih.gov). Feline genomic coding sequences of *COLQ* exon 1 and 2 from a healthy (wild-type) Sphynx cat were submitted to GenBank. Accession numbers are (GenBank: KT223392) for exon 1 and (GenBank: KT223393) for exon 2.

PCR and sequencing primers were designed using Primer3 [21]. Exonic genomic sequences were amplified and sequenced using primers from S1 Table. Exons were amplified individually or by pairs for each cat from 100 ng of their genomic DNA according to the manufacturers’ protocol, with Q-Bio Taq DNA Polymerase (Qbiogen MP Biomedicals Inc., Carlsbad, CA). Four hundred ng of each PCR amplicon were sent to GATC Biotech (GATC Biotech AG, Konstanz, Germany); there, they were purified and Sanger-sequenced in both forward and reverse directions. Electropherograms were manually inspected with Chromas Lite (Technelysium Pty Ltd, South Brisbane, Australia). Multiple alignments were performed using Multalin ([22]; http://multalin.toulouse.inra.fr/; identity matrix).

### Protein sequence comparisons and Structural prediction

Cat, human, mouse, cow, chicken, xenopus, zebrafish and fugu COLQ sequences were collected from Ensembl [www.ensembl.org; cat: (ENSFCAP00000010502.3), human: (ENSP00000373298.3), mouse: (ENSMUSP00000107658.2), cow (ENSBTAG00000040193), chicken (ENSGALG00000011202), xenopus (ENSXETG00000034209), zebrafish (ENSDARG00000019692), fugu (ENSTRUG00000015630)]. Multiple alignments were performed using Multalin ([22]; http://multalin.toulouse.inra.fr/; BLOSUM-62 and identity matrix). The predicted impact of missense mutations was assessed using Polyphen-2 [23], SNAP [24] and PROVEAN ([25]; provean.jcvi.org/seq_submit.php).

### Accession Numbers

Genomic coding sequences of *COLQ* exon 15 from healthy (wild-type) and affected (mutated) Sphynx cats (*Felis catus*) were submitted to GenBank. Accession numbers are (GenBank: KR049220) for the wild-type allele and (GenBank: KR049221) for the c.[1190G>A] mutated allele.

Feline genomic coding sequences of *COLQ* exon 1 and 2 from a healthy (wild-type) Sphynx cat were submitted to GenBank. Accession numbers are (GenBank: KT223392) for exon 1 and (GenBank: KT223393) for exon 2.

## Results

### A case of Devon Rex neuromuscular disorder in a Sphynx family

**Fig 1:**
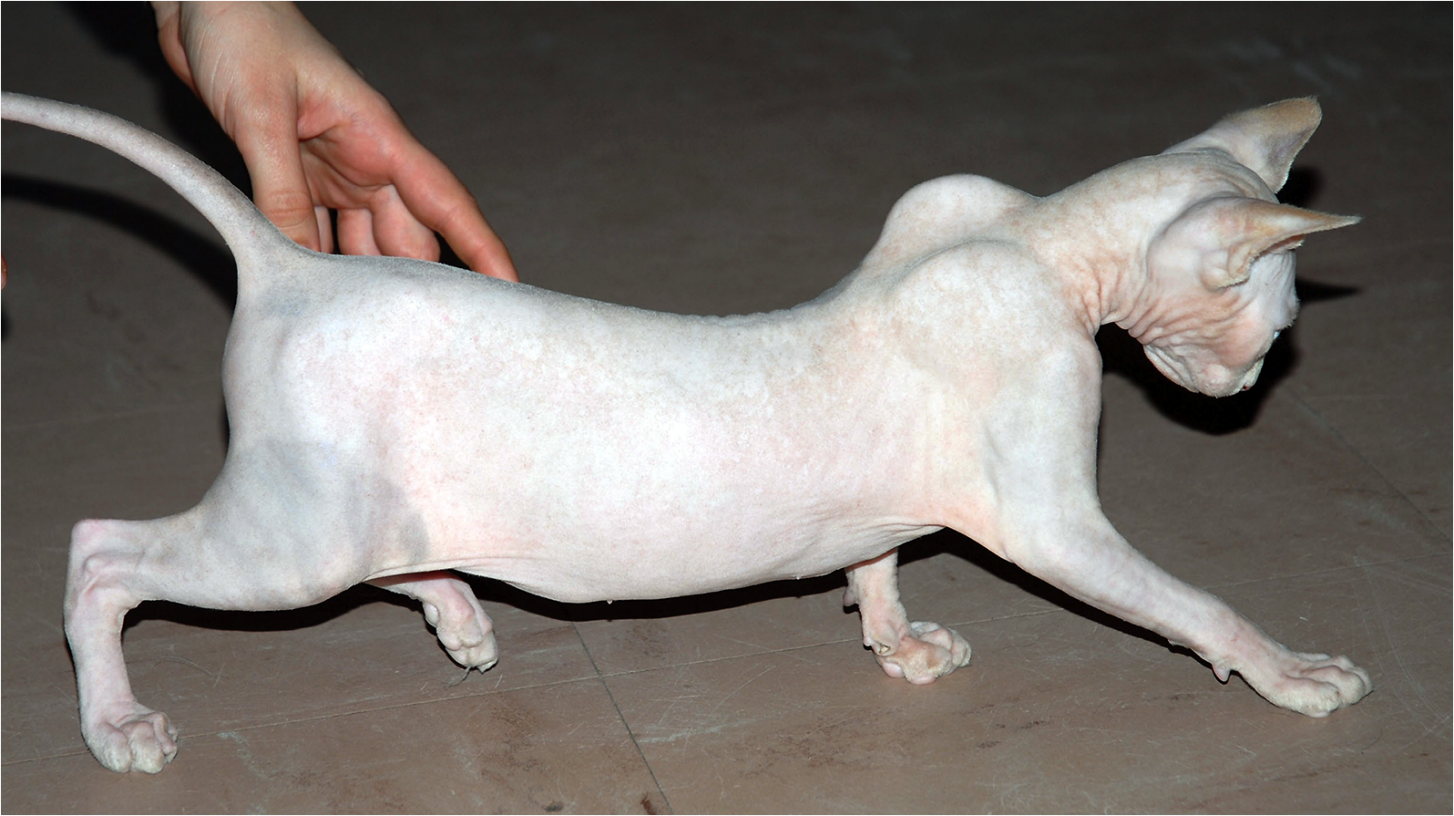
Congenital neuromuscular disorder in a Sphynx kitten. Picture of the four-month-old Sphynx female kitten presented at the Neurology clinics located at the Alfort School of Veterinary Medicine campus, in Maisons-Alfort, France. The kitten displayed a peculiar gait while walking, with ventroflexion of her neck. Note the marked dorsal protrusion of scapulae. No significant muscle atrophy was noticed.

**Fig 2:**
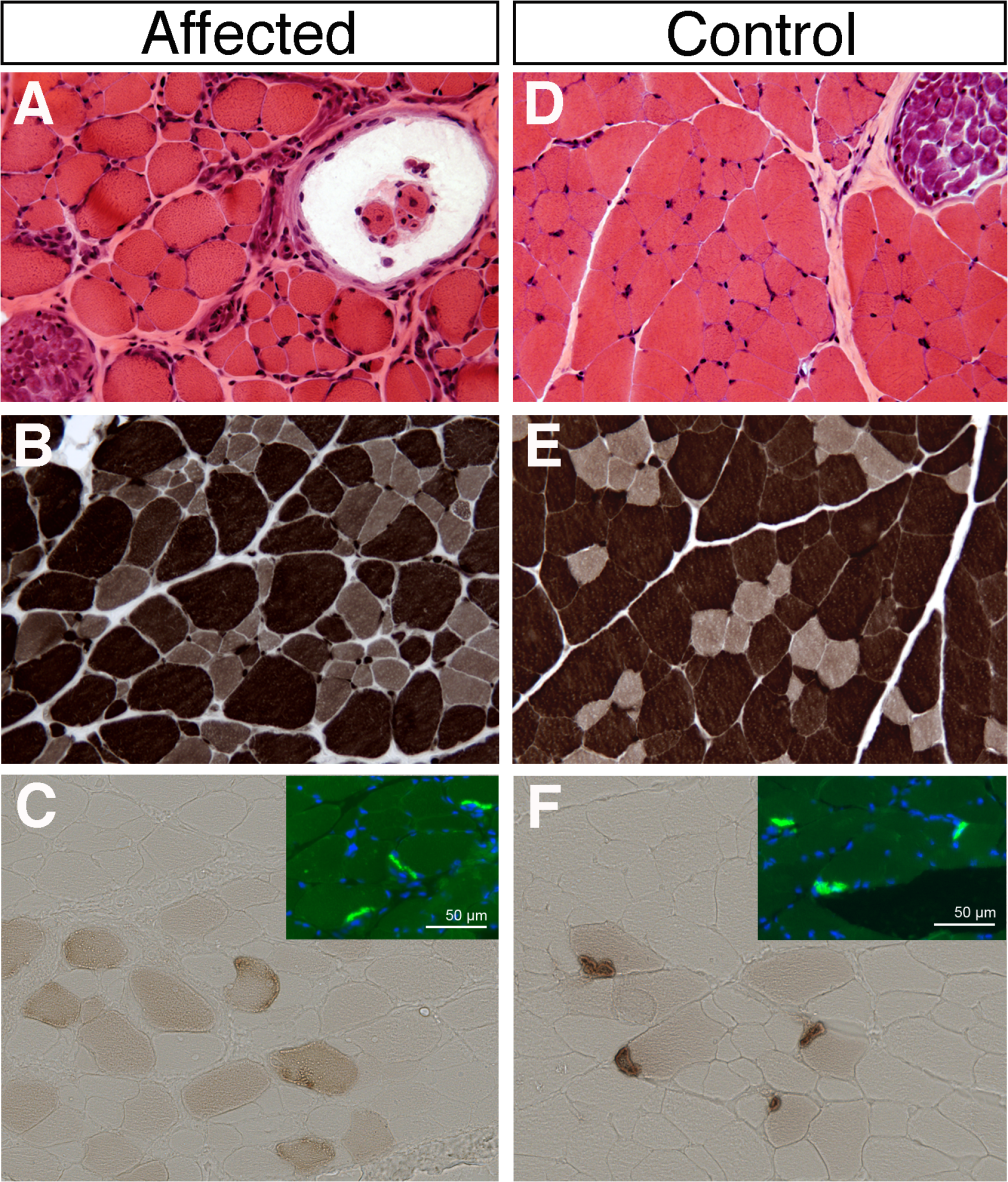
Histological and histochemical features of the disease. Cryosections (6 μm) of cervical muscle from a four-month-old affected Sphynx kitten (A-C) and from her healthy littermate (D-F). Haematoxylin-eosin staining (A, D) showed a wide range of fibre size with more rounded fibres surrounded by a thicker endomysium in the affected kitten (A) compared to the healthy control (D). No internal nuclei were present on both sections. Myosin adenosine triphosphatase isoforms (ATPase activity at pH 9.4, B and E) showed a slight increase in pale type-1 fibres in the affected kitten (B) compared to the healthy control (E) but no fibre-type aggregation. Motor end-plates were labelled with alpha-bungarotoxin (for acetylcholine receptors AchR; C and F insets; green) and esterase activity (for AchE; C, F; brown stain). Muscle nuclei were stained with Dapi (C and F insets; blue). Sections from the healthy littermate (F) showed a perfect colocalization of compacted AchR (green) and AchE (brown). In contrast, sections from the affected cat showed a faint, abnormally dispersed AchE staining in myofibres with normal clusters of AchR (C).

In 2007, a four-month-old female proband kitten from the Sphynx breed showing reduced activity and weakness was presented with her unique littermate (female) at the Neurological clinics located at the Alfort School of Veterinary Medicine in Maisons-Alfort, France. Onset of clinical signs had started a month before presentation, with reduced activity, weakness and slightly after, abnormal postures and locomotion. The affected kitten needed frequent resting periods when playing. During the clinical evaluation, no muscle atrophy was noticed; she however displayed a peculiar gait while walking, with shoulder blades held high and ventroflexion of the neck. At both rest or during walking, she displayed a marked dorsal protrusion of scapulae (Fig 1). After short playing sequences with her littermate, the affected female showed a “dog-begging” position, with front legs resting on a stair. Exercise was followed by stride shortening and limb tremors. The breeder also reported difficulties in taking and swallowing food, as well as occasional choking episodes while eating. During these acute sequences, food was placed on a raised platform and the breeder supervised its cat to prevent possible suffocation from swallowed food into the trachea. Neurological examination was normal in the affected female and her littermate, including normal responses of cranial and appendicular nerves. Electromyographic recording revealed a weak, slightly abnormal spontaneous activity within the tibialis anterior of the affected female, with normal velocities of tibial and ulnar nerves. Repetitive stimulation was not performed. Blood count, routine serum biochemical parameters and creatine kinase activity were all within reference ranges for the two littermates. Biopsies of the cervical, triceps brachii and biceps femoris muscles were performed in the two cats. Muscles from the healthy littermate displayed normal features on histological and histo-enzymological sections (Fig 2 and S2 Fig). By contrast, sections of the cervical muscle sampled from the affected female showed the most prominent abnormalities, including fibre size and shape variation; most of myofibres appeared round and atrophic or hypertrophic. Muscle spindles could be observed and were unremarkable, as were intramuscular nerves which density and myelin thickness seemed normal. Fibres were all well differentiated. No topographic aggregation for type-1 or type-2 fibres was noticed (Fig 2). Molecular components of NMJs were stained using a fluorescent alpha-bungarotoxin (postsynaptic acetylcholine receptor AchR) or acetylcholine esterase activity (synaptic AchE). On transverse muscle sections from the control cat, NMJs appeared normal with dense and compacted AchR clusters that colocalized with aggregate AchE activity; by contrast, NMJs of myofibres from the affected Sphynx littermate revealed an abnormal, dispersed AchE staining, with normal clustering of AchR (Fig 2).

The clinical signs reflected a global dysfunction of striated muscles, while the age of onset and the observed histopathological features both suggested that this proband was affected by the inherited neuromuscular disorder previously reported in the Sphynx breed [13].

**Fig 3:**
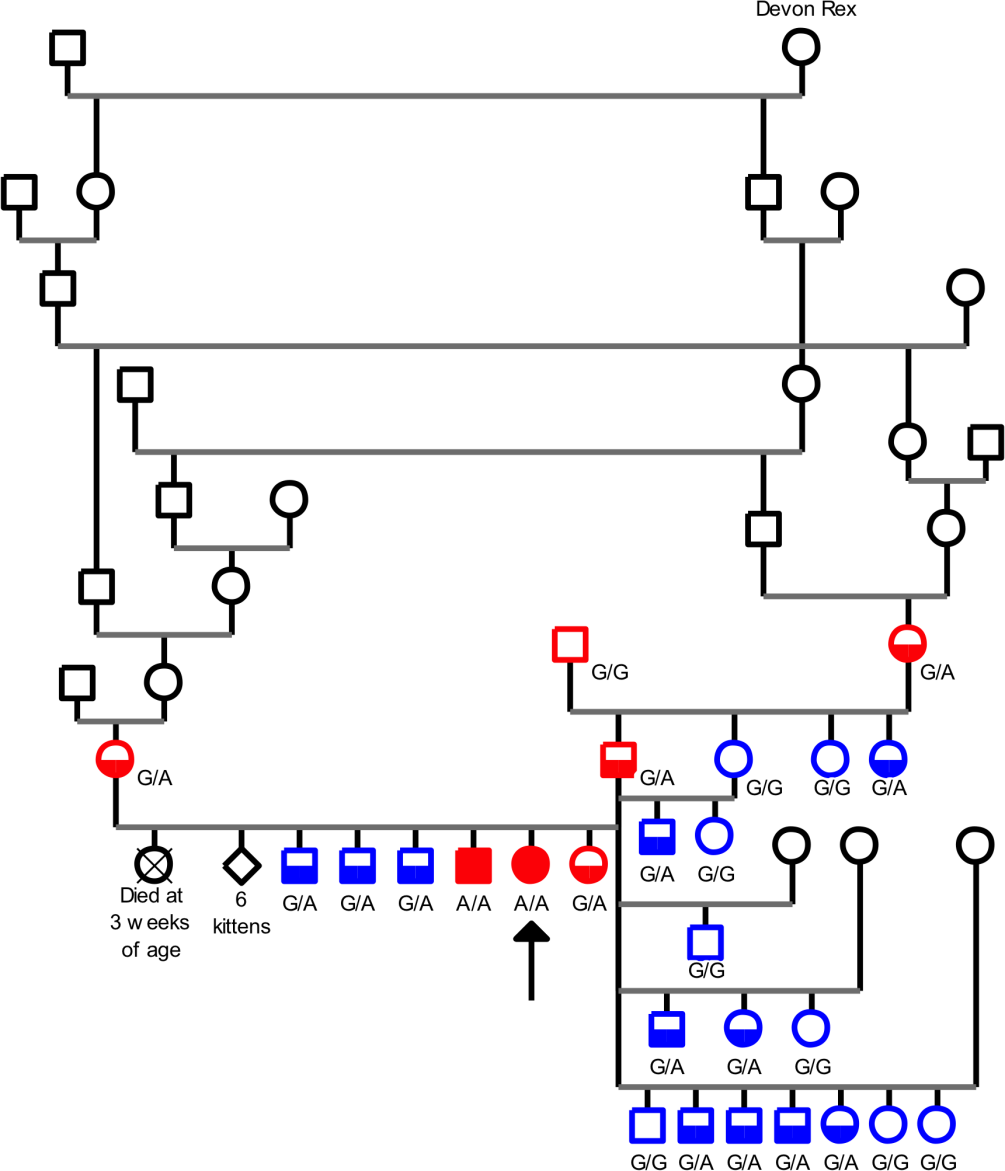
Pedigree-tree of a Sphynx cat family segregating a neuromuscular disorder. Circles represent females, squares represent males. Affected kittens are depicted with fully filled symbols and the proband shown with an arrow. Healthy carriers are depicted with two-toned symbols. Red symbols represent the seven cats from the nuclear family used to map the disease locus; blue symbols represent the four healthy siblings of the proband and the 16 healthy cats directly related to the sire; black symbols represent cats from the extended family. When available, result of the genotyping assay for the c.1190G>A variant is mentioned. The Devon Rex female born in 1990 and known by breeders to have several myopathy healthy carriers in her pedigree is shown (Devon Rex).

Both parents were healthy; they subsequently produced three other litters yielding one affected male (S1 Fig), nine unaffected cats and a female that died precociously from asphyxia at three weeks of age. Healthy parents and affected kittens supported the autosomal recessive inheritance pattern previously reported for this neuromuscular disorder (Fig 3; [13]). Pedigree examination of the two parents revealed ancestors from the Devon Rex breed, which had previously been identified as carriers of the Devon Rex neuromuscular disorder [15, 26]. In addition, some of these Devon Rex ancestors were shared by the sire and dam of the affected Sphynx cats reported in the present study (Fig 3).

To initiate the identification process of the molecular aetiology of this neuromuscular disorder, DNA was collected from relevant available cats: the two affected cats from two different litters (affected sib-pair), four of the nine unaffected sisters and brothers (including the healthy female littermate of the proband), sire and dam, and the two healthy paternal grandparents (Fig 3).

### Homozygosity mapping pointed out a unique locus on chromosome C2

**Fig 4:**
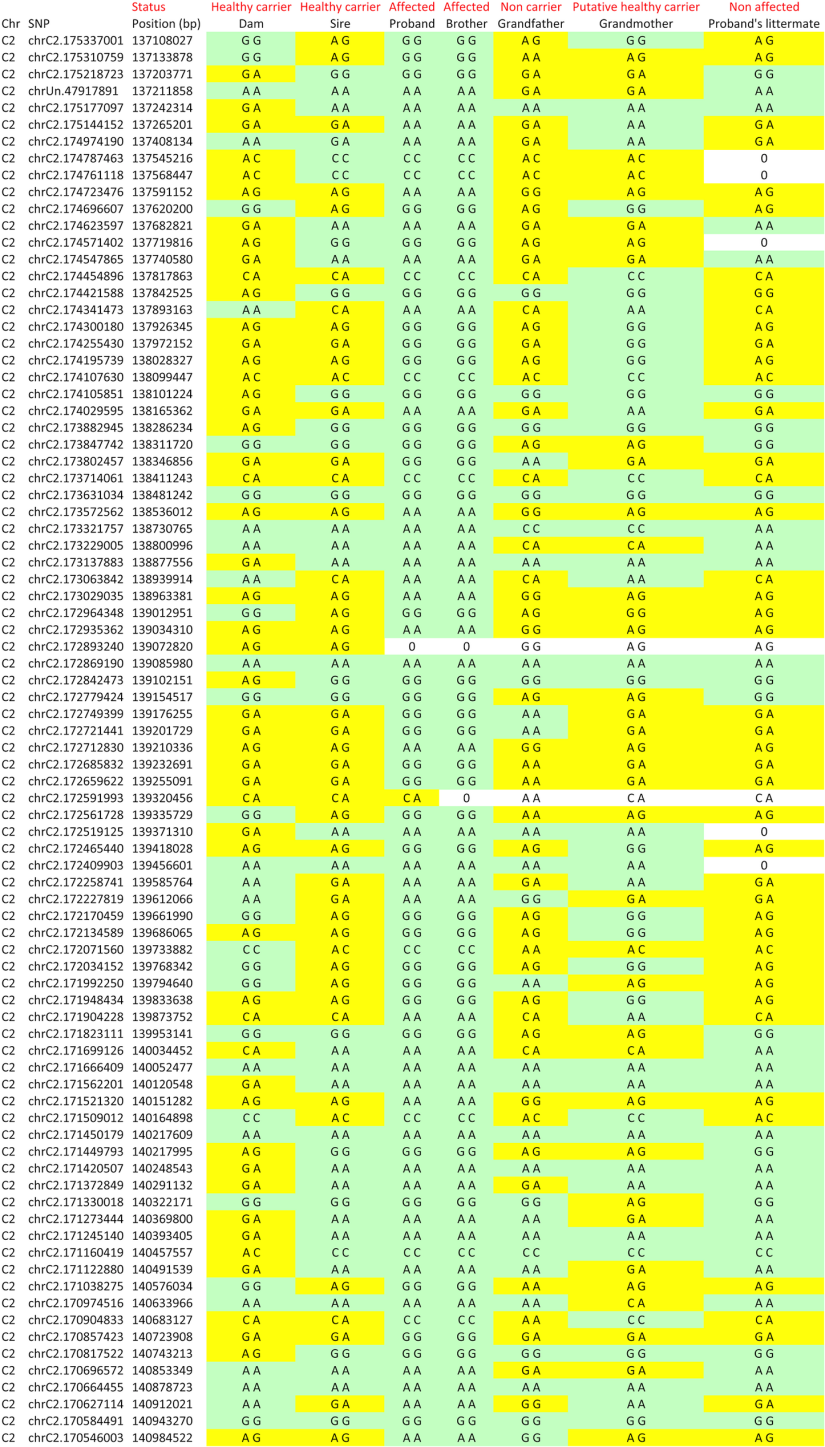
Genotypes for chromosome C2 candidate region. SNP genotypes for each cat were manually inspected in Excel to identify homozygous regions shared by the two affected cats. Only one region from chromosome C2 spanning from position 137108027 bp to position 140984522 bp (according to the updated felCat5 SNP manifest for the Illumina Feline 63k SNP genotyping array, [20]) was consistent with the highly-probable heterozygous status of the sire and dam, the non-homozygously mutated status of the proband’s healthy littermate and the inferred heterozygous status of the paternal grandmother. This region encompassed 3.9 Mb. Homozygosity for the allele shared by the affected sib-pair is shown in light green. Heterozygosity or homozygosity for the opposite allele is shown in yellow. Missing genotypes are noticed 0. Chr: chromosome. bp: base pairs.

Genome wide association studies using single nucleotide polymorphism (SNP) arrays have proved a successful approach to identify recessive mutations in felines when cohorts of several affected cases and controls are available [27, 28]. In the present study, only two affected cats were available. To circumvent this limitation, we used an homozygosity mapping strategy in our nuclear family. The affected sib-pair, the healthy littermate of the proband, their two healthy parents and the two paternal grandparents were genotyped using the Illumina Feline 63k SNP array, among which 61,705 SNPs yielded usable results. The seven cats had genotyping rates > 95% and were all conserved for the analysis. Genotypes for each chromosome were manually inspected to identify homozygous regions shared by the two affected cats. Six regions located on chromosomes A3, B3, C1, C2, D1 and E1 were identified (S2 Table). Only one region from chromosome C2 was consistent with the highly probable heterozygous status of the two parents, the non-homozygously mutated status of the proband's healthy littermate and the inferred heterozygous status of the paternal grandmother; this region encompassed 3.9 Mb (Fig 4). Comparison of genes within or in the vicinity of the identified region with a reference list of genes possibly causing neuromuscular disorders when mutated [29] revealed *COLQ* as the closest presumptive candidate gene (chromosome C2: 133071353–133148521 bp, GenBank ID: 101093134, Felis_catus 8.0 GenBank assembly, annotation release 102). According to the felCat5 SNP manifest based on the Felis_catus 6.2 GenBank assembly [20], the identified candidate region on chromosome C2 was lying 3.9 Mb from *COLQ*. Inconsistencies between versions of the feline genome assembly, on the one hand, and between genomic locations of SNPs from the Illumina 63k array and versions of the genome assembly, on the other hand [20], led us to still consider *COLQ* as a good candidate gene.

### A missense mutation in *COLQ* segregates in the Sphynx family

The *COLQ* feline partial sequence was collected from Ensembl (ENSFCAG00000011304), and the missing exon 1 was extracted from the trace archive databases for *Felis catus* (WGS; http://blast.ncbi.nlm.nih.gov) by aligning raw sequences with exon 1 sequence of the human *COLQ* gene (www.ensembl.org; ENST00000383788). The complete feline sequence was thus used to design 13 sets of primers covering the whole coding sequence (S1 Table). We successfully amplified the 17 exons of the gene in the two affected cats, sequenced them and performed pair-wise base-to-base comparisons using Multalin. A unique variant was revealed between the two affected Sphynx cats and the Abyssinian feline reference sequence. This c.1190G>A non-synonymous variant located within exon 15 produced cysteine to tyrosine replacement at position 397 in the protein, predicted by PROVEAN (score = -3.797), PolyPhen-2 (score = 0,998) and SNAP (expected accuracy = 63%) to be a deleterious variant. The two affected Sphynx cats were homozygous for the *A* allele, which was consistent with the recessive mode of inheritance of the aforementioned disease. In parallel, sequences of additional genes from the candidate region were obtained and yielded no deleterious variant (S3 Table).

In a second series of experiments, available members of our family were genotyped for the c.1190G>A *COLQ* variant. In addition to the two affected cats, this validation cohort included eight Sphynx cats from the family (Fig 3). The two parents, the paternal grandmother and the four normal siblings of the proband were all heterozygous *G/A*; the paternal grandfather was homozygous *G/G* (Table 1). Additionally, we recruited 16 Sphynx cats directly related to the sire (three sisters of the sire and 13 half siblings of the proband, born to the sire and to four different females, Fig 3) and an affected Devon Rex cat born in 2013 in Switzerland (S1 Fig). The 16 healthy Sphynx cats were either heterozygous *G/A* (n = 8) or homozygous *G/G* (n = 8); the affected Devon Rex was homozygous for the *A* allele (Table 1). Finally, we genotyped 306 control cats from 14 breeds, including 14 Devon Rex—none of them carried the *A* allele for the c.1190G>A variant—and assessed the percentage of cats carrying the c.[1190G>A] *COLQ* allele in a subpanel of 81 control Sphynx cats not linked to the family and excluding first-degree relatives and found it to be 3.7% (Table 2).

**Table 1:** Genotypes for the c.1190G>A variant in affected cats and unaffected relatives.

Individuals	*G/G*	*G/A*	*A/A*	Total
Affected Sphynx (n = 2)	0	0	2	2
Affected Devon Rex (n = 1)	0	0	1	1
Sphynx healthy carrier parents (n = 2)	0	2	0	2
Sphynx putative healthy carrier paternal grandmother (n = 1)	0	1	0	1
Sphynx healthy paternal grandfather (n = 1)	1	0	0	1
Sphynx healthy brothers and sisters of the proband (n = 4)	0	4	0	4
Sphynx healthy sisters of the sire (n = 3)	2	1	0	3
Sphynx half siblings of the proband (n = 13)	6	7	0	13
Total	9	15	3	27

**Table 2:** Genotypes for the c.1190G>A variant in 14 breeds of cats.

Breed	*G/G*	*G/A*	*A/A*	Total	c.[1190G>A] percentage
Control Sphynx cats	78	3	0	81	3.7%
Control Devon Rex cats	14	0	0	14	0%
Siamese & Oriental SH and LH	22	0	0	22	0%
Persian	20	0	0	20	0%
British SH and LH	20	0	0	20	0%
Birman	27	0	0	27	0%
Ragdoll	10	0	0	10	0%
Norwegian Forest	22	0	0	22	0%
Maine Coon	21	0	0	21	0%
Chartreux	16	0	0	16	0%
Bengal	15	0	0	15	0%
Abyssinian	7	0	0	7	0%
Russian Blue	4	0	0	4	0%
Outbred domestic shorthair cats	27	0	0	27	0%
Total	303	3	0	306	

SH: shorthair, LH: longhair

Hence the 100% concordance observed between the autosomal recessive mode of inheritance of the disease and the genotypes of cats from and outside our pedigree, corroborated our hypothesis of full association between the c.1190G>A variant in *COLQ* and the neuromuscular disorder reported in Sphynx and Devon Rex cats.

### The associated allele is predicted to code a COLQ mutated protein causing human synaptic CMS

**Fig 5:**
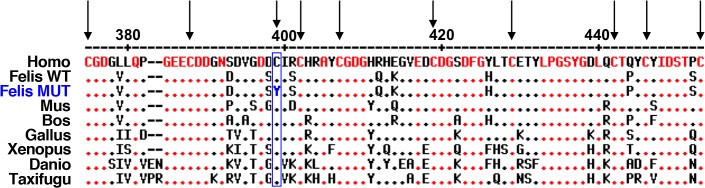
Wild-type and mutant C-terminal domains of COLQ proteins. Alignment of partial protein sequences of COLQ, translated from the c.[1190G>A] mutated allele identified in affected Sphynx and Rex Devon cats (Felis MUT) or wild-type alleles reported in human (Homo), mouse (Mus), cow (Bos), chicken (Gallus), xenopus (Xenopus), zebrafish (Danio), fugu (Taxifugu) and cat (Felis WT). The cysteine-rich domain of the C-terminal end of the protein starts with amino acid number 375 in human and cat proteins and ends with amino acid number 451 (according to [10]). Human COLQ sequence was used as the reference sequence. Conserved residues are written in red within the reference sequence and represented by red dots in other sequences. Dashes represent deletions. Arrows point out the ten conserved cysteine residues. Cysteine 397, mutated in affected cats (C397Y), is surrounded in blue.

To evaluate the functional importance of the C397 residue, we aligned the feline COLQ protein sequence with the COLQ sequences of seven vertebrates (Fig 5 and S2 Fig). We found a 87% identity between feline and human COLQ proteins and a full conservation of the C397 residue between the seven COLQ sequences. This high level of conservation was previously reported for the 10 essential cysteines of the cysteine-rich domain of COLQ C-terminal region, spanning from residues 375 to 451 in the human protein [10–12]. In addition, the feline C397 residue of COLQ best aligns with C397 of its human ortholog (Fig 5 and S2 Fig), found to be mutated in human patients affected by endplate AChE deficiency (i.e. synaptic CMS) [10–12].

Altogether, these convergent results strongly suggest that the C397 residue is essential for AChE activity at the neuromuscular junction. The observation that its alteration in humans and cats leads to a phenotype fully compatible with a loss-of-function mutation in COLQ, emphasizes that the identified recessive mutation is causative of the neuromuscular disorder observed in Sphynx and Devon Rex cats.

## Discussion and Conclusions

### Characteristics of the neuromuscular disorder of Sphynx and Devon Rex cats are consistent with a congenital myasthenic syndrome

Spasticity [14], hereditary myopathy [15] of Devon Rex cats or muscular dystrophy [30] of Devon Rex and Sphynx cats have been reported in these two related breeds for many years, yet the molecular etiology of these related neuromuscular disorders and its precise nosological classification remained to be elucidated. SNP-based homozygosity mapping followed by a candidate gene approach was proved successful in the identification of recessive mutations using a unique affected sib-pair both in humans [31] and dogs [7]. Hence we used a sib-pair of affected Sphynx cats and identified a c.1190G>A variant in the exonic sequence of *COLQ*, predicted *in silico* to yield a C397Y deleterious mutation. Using structural analysis and *in vitro* assays, five human missense mutations including C397S, C400Y, Y404D, C405F and G423V were shown to specifically disrupt the interaction of COLQ with MuSK and other proteins of the basal lamina [10–12]. The absence in an affected Sphynx cat of AchE aggregates at the NMJ highly supports that the identified c.[1190G>A] allele yields a mutated COLQ protein with deficient protein-protein interactions [10] and thus confirms a pivotal role of this residue in the COLQ-dependent, synaptic clustering of AchE.

By identifying an homozygote, affected Devon Rex cat, we strengthened causality of the mutation and argued that the c.[1190G>A] allele would be a founder unique mutation predisposing the two related Sphynx and Devon Rex breeds to the same neuromuscular disorder. An haplotype analysis revealing identity-by-descent of the chromosomal region encompassing *COLQ* in cats from the two breeds would be necessary to confirm this plausible hypothesis.

In the first description of the disease, six related Devon Rex cats exhibiting a slow progressive congenital muscle disease were diagnosed [15]. Affected cats showed prominent ventroflexion of the head and neck, head bobbing, protrusion of the scapulae, megaoesophagus and pharyngeal weakness. Malik and his collaborators described significant lesions in muscle. Muscle biopsies showed variation in cross sectional area, with rounded fibres, occasional degenerating or segmenting fibres and increased subsarcolemmal or internal nuclei. Dorsal cervical and proximal forelimb muscles were more affected than proximal hindlimb and distal limb muscles. Peripheral nerves, spinal cord and brain were grossly and histologically normal. Electrodiagnosis performed in one cat showed a within-range response to repetitive supramaximal nerve stimulation. These muscular signs associated with an absence of neurogenic features in muscle biopsies prompted authors to conclude to a primary muscular dystrophy ([15], S4 Table). In the second study, two Sphynx cats and a Devon Rex cat were investigated. Disease in the Devon Rex showed common features with the first description of Malik and his collaborators, including clinical presentation, excessive variability in myofibre size, internal nuclei, occasional necrotic fibres and several regenerating fibres. Histological modifications were more pronounced in the cervical muscle than in the triceps muscle. Sphynx cases showed only mild myopathic changes including excessive variability in myofibre size, infrequent centralized nuclei and type-1 fibre predominance. Interestingly, repetitive supramaximal nerve stimulation revealed a decremental response in one of the two Sphynx cats ([13], S4 Table).

In the present report, histological examination of muscle biospies from the proband showed excessive variability in myofibre size with no obvious fibre-type predominance (Fig 2 and S4 Table). In addition, we revealed a NMJ defect in affected myofibres, characterized by the absence of AchE clustering.

Although not strictly identical, clinical features of all reported feline cases were concordant (S4 Table). Also, variability in histopathological features seems to mimic the unspecificity of some muscle remodeling, such as type-1 fibre predominance, well-described in only some of the human patients affected by CMS due to *COLQ* mutations [32]. Interestingly, some CMS patients display a limb-girdle phenotype with a predominant weakness of proximal muscles and no decremental response to repetitive nerve stimulation [32], as observed in cats. Compared to human patients, affected cats are subject to chocking episodes due to laryngeal obstruction during feeding. Because they are quadrupeds, their larynx and esophagus are in a more horizontal position, which might explain the high frequency of upper airway obstructions if not fed in raised dishes (S4 Table).

In conclusion, clinical, histological and electrophysiological features of the neuromuscular disorder segregating in Sphynx and Devon Rex cats are consistent with a CMS. In particular, absence of systematic esterase activity clustering suggests an impaired anchoring of AchE at the NMJ. In agreement with the homozygous status of three affected cats for a deleterious *COLQ* mutation, we propose to rename the muscular dystrophy of the Devon Rex and Sphynx cats, the CMS of Devon Rex and Sphynx cats. Variability in clinical signs and histopathological features between affected cats may result from modifying alleles of genes involved in functional networks such as neuromuscular transmission or muscle homeostasis, yet to be investigated in the future.

### The *COLQ* mutation is restricted to Sphynx and Devon Rex cats

Genotyping of 211 cats from 12 non-Sphynx and non-Devon Rex breeds for the *COLQ* c.[1190G>A] allele failed to identify carriers (Tables 1 and 2). Devon Rex cats were initially bred in Great Britain in 1960 and recognized by the GCCF (Governing Council of the Cat Fancy) in 1967 [33]. The Devon Rex is a shorthaired, curly-coated cat exhibiting a wavy silky coat and a peculiar morphology of the head with large eyes and ears. The Devon Rex coat mutation appeared spontaneously and was first seen in Buckfastleigh, Devon, UK [33]. In 1960, a feral curly-coated tomcat sired a domestic shorthair female who gave birth to normal kittens and to a curly-coated male who was domesticated and called Kirlee. Kirlee was the first Devon Rex cat and the common ancestor to all Devon Rex cats. Before official recognition of the breed, Devon Rex were mated with Cornish Rex, another curly-coated breed, without giving birth to curly-coated kittens, showing the distinct origin of the two curly mutations [33, 34]. Several other breeds were included in the genetic pool of the Devon Rex breed, including Persian, Siamese and British Shorthair (www.pawpeds.com). The first cases of muscle weakness were described in the breed in 1974 [15]. Pedigree analysis revealed that second-degree relatives of Kirlee gave birth to affected kittens, highlighting the presence of the deleterious mutation since the beginning of the breed [26]. The autosomal recessive inheritance pattern of the condition was recognized in 1992 [14].

The Sphynx breed is an almost hairless feline breed, which was first recorded in Canada in 1966 [16, 33]. This breed is related to the Devon Rex [35, 36]. The first hairless kittens were born to outbred domestic shorthair cats in Canada and USA. To enlarge the genetic pool of this new breed, several outcrosses were made using various breeds and especially the Devon Rex. Indeed, Sphynx and Devon Rex are both characterized by distinct mutations in the *Keratin 71* gene and compound heterozygous F1 kittens are often hairless [16]. As a congenital neuromuscular disorder similar to the Devon Rex condition was described in the Sphynx breed, a common origin for the disease was suspected [13], confirmed by our present results.

In our feline cohort, the c.[1190G>A] disease mutation was restricted to Sphynx and Devon Rex. Notably, none of the 22 Siamese/Oriental short/longhaired, of the 20 Persian/Exotic cats, and of the 20 British Shorthair/Longhair cats from our cohort carried the mutation. Additionally, the two affected Sphynx kittens and healthy carriers from our family were related to a female Devon Rex born in 1990 and known by breeders to have several healthy carriers of the disease in her ancestors (Fig 3, [26]). Therefore, we can hypothesize that the Sphynx and Devon Rex *COLQ* mutation was present at the beginning of the Devon Rex breed and spread across the Sphynx breed thanks to outcrosses performed during 70’s to 90’s. In our family, the mutation would have been transmitted through the maternal and paternal grandmothers breeding lines.

### The *COLQ* deficient cat: a novel large animal model for synaptic CMS

Since the publication of the first human *COLQ* mutation in 1998 [37], *COLQ*-deficient engineered mice have been generated to study endplate AChE deficiency and to model human synaptic CMS (www.informatics.jax.org). To date, they remain relevant models to decipher the pathophysiology of—and test new therapies for—CMS and myasthenia gravis (autoimmune myasthenic syndrome) [38, 39]. However, limitations of murine models in preclinical studies for neuromuscular disorders have been identified, as shown for gene or cellular therapy of Duchenne muscular dystrophy [40, 41]. As a consequence, there is a tremendous demand for more accurate animal models in the neuromuscular field, providing a more realistic set of physiological and outcome estimates. To the best of our knowledge, the neuromuscular disorder characterized here at the molecular level, represents the first feline CMS model spontaneously mutated in *COLQ*. With Labrador Retrievers recently identified with a *COLQ*-associated CMS [7], the feline CMS described here could represent a complementary, spontaneously affected large animal model mimicking the orthologous human condition. These models would be highly appreciated for the purpose of comparative analyses and to fill the gap between rodent models and humans in the evaluation of future therapeutic strategies.

Of note, ethical issues related to the use of a companion animal in experimental procedures, the cost of husbandry and the specialized care will have to be carefully balanced with scientific data. Consent of owners and ethical committees will be a prerequisite to any preclinical trial performed in dogs or cats.

In conclusion, our results describe the first recessive *COLQ* loss-of-function allele described in felines. We propose that the c.[1190G>A] allele, which is easily detectable with a genetic test and associated with the neuromuscular disorder that was previously named spasticity, myopathy or muscular dystrophy of the Sphynx and Devon Rex, defines the first model of congenital myasthenic syndrome in cats. Identification of the mutation underlying the neuromuscular disorder in Sphynx and Devon Rex cats will allow the prevention of matings at risk and avoid the birth of disabled kittens. The DNA test resulting from this study is available to the community and will help breeders identify healthy carriers in their breeding stocks.

Identifying this neuromuscular disorder as a CMS also opens the opportunity to further evaluate in affected cats the efficiency of ephedrine treatment, successfully used to improve health conditions in *COLQ*-deficient patients [32, 42].

## Supporting Information

S1 Fig Affected Sphynx male and Devon Rex female showing an atypical resting posture. Note the atypical resting position of these two affected 18-month-old Sphynx male (A) and 15 month-old Devon Rex female (B). Both cats rely on the edge of their bed to support their head, open their rib cage and breathe more easily.
(TIF)

S2 Fig Histological features of the disease. Cryosections (6 μm) of cervical muscle from a four-month-old affected Sphynx kitten (A-C) and from her healthy littermate (D-F). Modified Gomori trichrome staining (A, D) showed normal mitochondrial distribution and myelinated nerves in the affected kitten (A). Oil red O staining for lipids (B, E) showed type-1 fibres with more lipid droplets than type-2 fibres and revealed no difference between the two sections. Reduced nicotinamide adenine dinucleotide dehydrogenase-tetrazolium reductase staining (C, F) confirmed normal mitochondrial distribution and absence of myofibrillar disruption. Normal dark peripheral areas of clustered mitochondria were present in sections from both kittens.
(TIF)

S3 Fig Alignment of the predicted mutant COLQ protein with full-length wild-type orthologs. Alignment of protein sequences of COLQ, translated from the c.[1190G>A] mutated allele identified in affected Sphynx and Rex Devon cats (FelisMUT) or the wild-type alleles reported in human (Homo), mouse (Mus), cow (Bos), chicken (Gallus), xenopus (Xenopus), zebrafish (Danio), fugu (Taxifugu) and cat (FelisWT). Human COLQ sequence was used as the reference sequence. Conserved residues are written in red within the reference sequence and represented by red dots in other sequences. Dashes represent deletions.
(TIFF)

S1 Table *COLQ* PCR and sequencing primers. Sequences and PCR temperatures from the intronic primers that were used to amplify and sequence the 17 *COLQ* coding exons.
(DOCX)

S2 Table Homozygous regions shared by the two affected cats. SNP genotypes for each cat were manually inspected with Excel to identify homozygous regions shared by the two affected cats. Only one region from chromosome C2 was consistent with the highly-probable heterozygous status of the sire and dam, the non-homozygously mutated status of the proband’s healthy littermate and the inferred heterozygous status of the paternal grandmother. Homozygosity for the allele shared by the affected sib-pair is shown in light green. Heterozygosity or homozygosity for the opposite allele is shown in white (chromosomes A3, B3, C1, D1 and E1) or in yellow (chromosome C2). Missing genotypes are noticed 0. Chr: chromosome. bp: base pairs.
(XLSX)

S3 Table Genes located in the critical region and excluded as candidate genes. Ensembl gene identifications are provided (www.ensembl.org). The description of the genes was obtained from the GeneCards database (www.genecards.org/). Expression data (mouse and human) were extracted from BioGPS (http://biogps.org). Mouse knockout data were extracted from the Mouse Genome Informatics (MGI) database (www.informatics.jax.org). Prediction of functional effect of SNP was performed using Polyphen2 (http://genetics.bwh.harvard.edu/pph2/index.shtml).
(XLSX)

S4 Table Disease features reported in the literature and in this study. Clinical, histological, biochemical, and electrophysiological features of the neuromuscular disease reported in Devon Rex and Sphynx cats in the literature and in this study.
(DOCX)
