# Supplementary figures and images for: A COLQ Missense Mutation in Sphynx and Devon Rex Cats with Congenital Myasthenic Syndrome
Source: PLoS One. 2015 Sep 1;10(9):e0137019. doi: 10.1371/journal.pone.0137019 (PMC4556666; doi:10.1371/journal.pone.0137019)

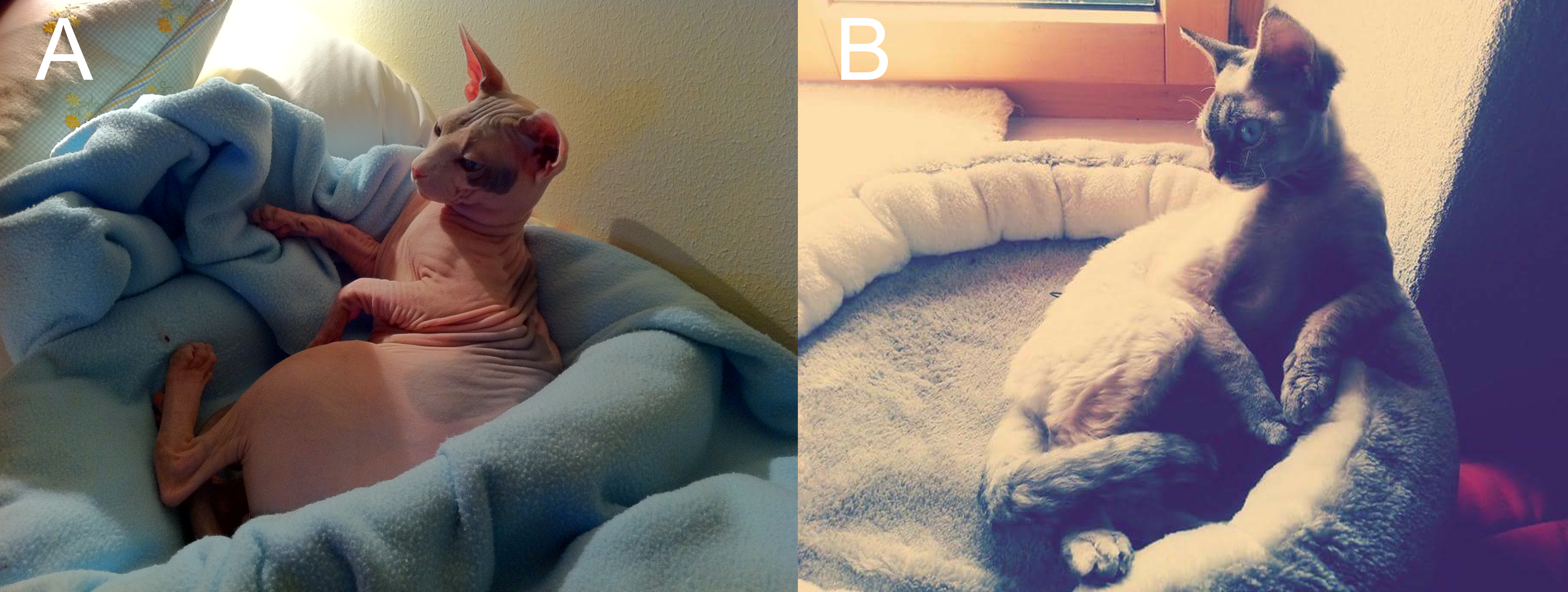

Supplement: S1 Fig — Note the atypical resting position of these two affected 18-month-old Sphynx male (A) and 15 month-old Devon Rex female (B). Both cats rely on the edge of their bed to support their head, open their rib cage and breathe more easily. (TIF) [file pone.0137019.s001.tif]

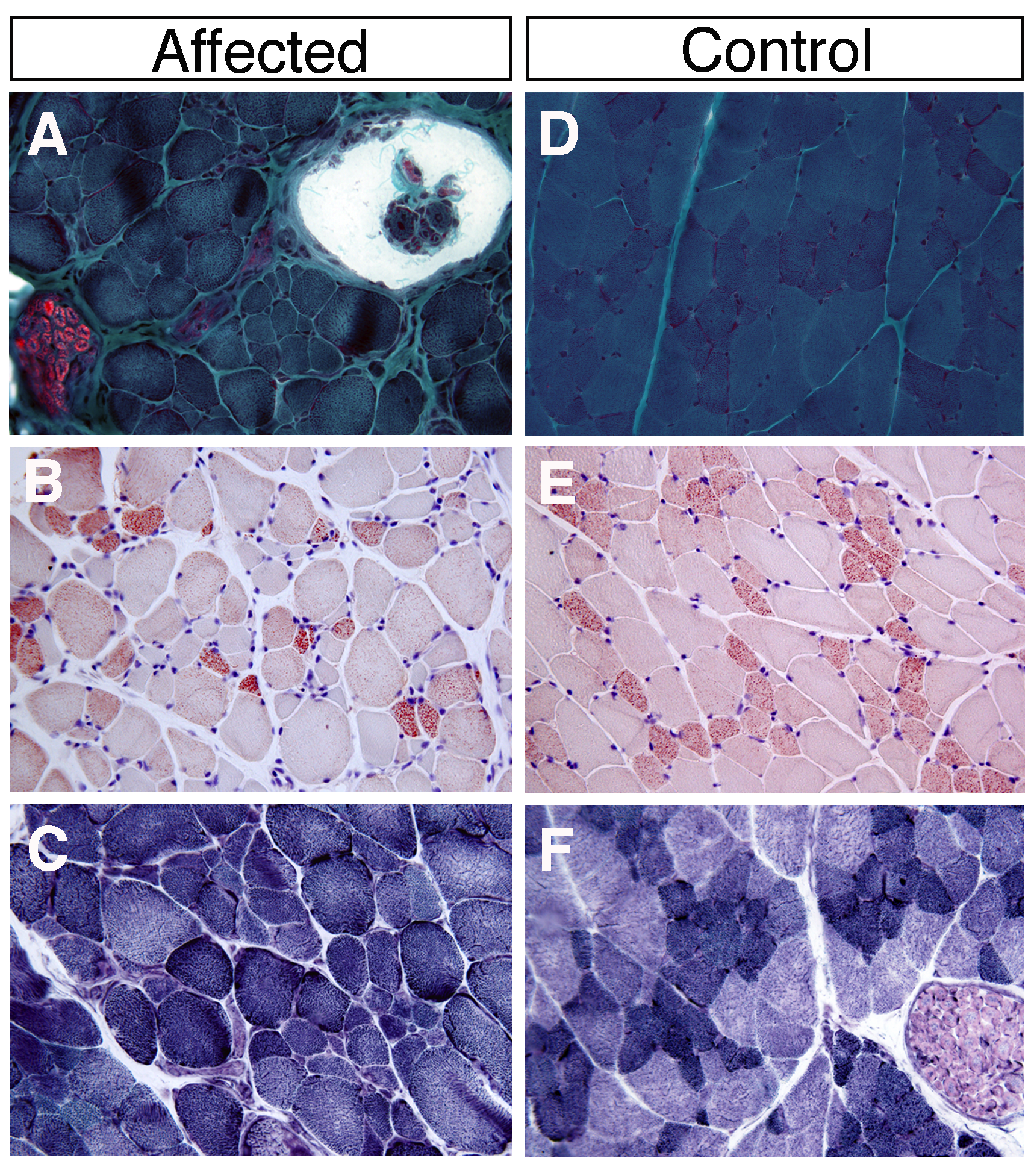

Supplement: S2 Fig — Cryosections (6 μm) of cervical muscle from a four-month-old affected Sphynx kitten (A-C) and from her healthy littermate (D-F). Modified Gomori trichrome staining (A, D) showed normal mitochondrial distribution and myelinated nerves in the affected kitten (A). Oil red O staining for lipids (B, E) showed type-1 fibres with more lipid droplets than type-2 fibres and revealed no difference between the two sections. Reduced nicotinamide adenine dinucleotide dehydrogenase-tetrazolium reductase staining (C, F) confirmed normal mitochondrial distribution and absence of myofibrillar disruption. Normal dark peripheral areas of clustered mitochondria were present in sections from both kittens. (TIF) [file pone.0137019.s002.tif]

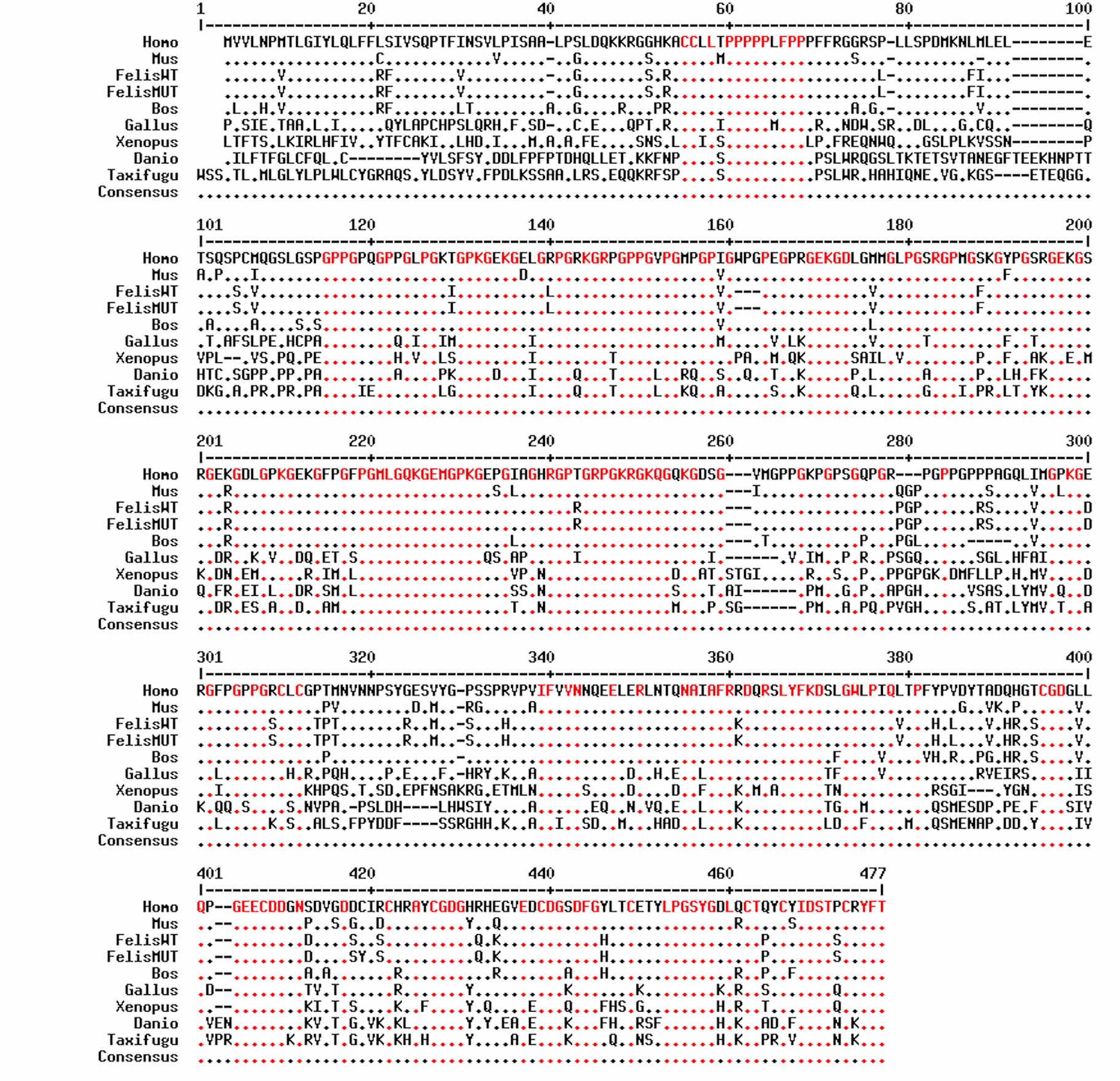

Supplement: S3 Fig — Alignment of protein sequences of COLQ, translated from the c.[1190G>A] mutated allele identified in affected Sphynx and Rex Devon cats (FelisMUT) or the wild-type alleles reported in human (Homo), mouse (Mus), cow (Bos), chicken (Gallus), xenopus (Xenopus), zebrafish (Danio), fugu (Taxifugu) and cat (FelisWT). Human COLQ sequence was used as the reference sequence. Conserved residues are written in red within the reference sequence and represented by red dots in other sequences. Dashes represent deletions. (TIFF) [file pone.0137019.s003.tiff]
