# Supplementary material for: A COLQ Missense Mutation in Sphynx and Devon Rex Cats with Congenital Myasthenic Syndrome
Source: PLoS One. 2015 Sep 1;10(9):e0137019. doi: 10.1371/journal.pone.0137019 (PMC4556666; doi:10.1371/journal.pone.0137019)
Supplement: S1 Table — Sequences and PCR temperatures from the intronic primers that were used to amplify and sequence the 17 COLQ coding exons. (DOCX) [file pone.0137019.s004.docx]

**S1 Table. *COLQ* PCR and sequencing primers**

| Exon(s) | Primers | Primer sequence | Tm | PCR product |
| --- | --- | --- | --- | --- |
| *COLQ* exon1 | Exon1F | GAG AGG CGC AAA CAG CAG | 58°C | 374 bp |
|  | Exon1R | GAC AGC TCG GGT GAC AGG |  |  |
| *COLQ* exon2 | Exon2F | ctg ggg gag ggc tac atc | 56°C | 401 bp |
|  | Exon2R | tca agt gcg ggg aag ttg |  |  |
| *COLQ* exon3 | Exon3F | cac cca ctg ggg tgt cag | 60°C | 366 bp |
|  | Exon3R | agc cgg agg tga gct agg |  |  |
| *COLQ* exons 4 and 5 | Exons4-5F | tgc tag agg agc cca acg | 56°C | 544 bp |
|  | Exons4-5R | cgt gca atg ctg aca gaa c |  |  |
| *COLQ* exon 6 | Exon6F | tca ctg gag ggt ggg atg | 58°C | 247 bp |
|  | Exon6R | cat gtt ctg ata cca cat gaa cc |  |  |
| *COLQ* exons 7 and 8 | Exons7-8F | ctt gcc cat gat ttt cct g | 56°C | 591 bp |
|  | Exons7-8R | tgg gcc tgt ctt ctg ctc |  |  |
| *COLQ* exons 9 and 10 | Exons9-10F | cgg gca agt cac ctg aag | 56°C | 527 bp |
|  | Exons9-10R | aac ctt cca cga cga aac c |  |  |
| *COLQ* exon 11 | Exon11F | gtc cgg aac gat cca gtg | 58°C | 296 bp |
|  | Exon11R | gct gat tgg ctc cac ctg |  |  |
| *COLQ* exon 12 | Exon12F | gga gtt tgg ggg tca agg | 58°C | 335 bp |
|  | Exon12R | ggc aga cct ctg ctg tgc |  |  |
| *COLQ* exon 13 | Exon13F | ccc agg gcc tac cct aag | 56°C | 448 bp |
|  | Exon13R | cca aat gga caa ctt ggt ttc |  |  |
| *COLQ* exons 14 and 15 | Exons14-15F | acc agc cca agg atg gag | 56°C | 821 bp |
|  | Exons14-15R | ttg gag atc acc cca acc |  |  |
| *COLQ* exon 16 | Exon16F | aga ggg tgc atg ctc tgg | 58°C | 374 bp |
|  | Exon16R | gag cag gga cca tgt gtt g |  |  |
| *COLQ* exon17 | Exon17F | ggt ggc ctc aga aca tgc | 58°C | 304 bp |
|  | Exon17R | tag cag caa ggg ctc gtc |  |  |

F: forward. R: reverse. Tm: melting temperature. bp: base pairs.
