# Supplementary material for: A COLQ Missense Mutation in Sphynx and Devon Rex Cats with Congenital Myasthenic Syndrome
Source: PLoS One. 2015 Sep 1;10(9):e0137019. doi: 10.1371/journal.pone.0137019 (PMC4556666; doi:10.1371/journal.pone.0137019)
Supplement: S4 Table — Clinical, histological, biochemical, and electrophysiological features of the neuromuscular disease reported in Devon Rex and Sphynx cats in the literature and in this study. (DOCX) [file pone.0137019.s007.docx]

**S4 Table. Phenotype of affected Sphynx and Devon Rex cats reported in the literature and in this study**

| Study | Cat | Age of onset | Clinical features | Biochemical features | EMG features | Histological features | Other features |
| --- | --- | --- | --- | --- | --- | --- | --- |
| Robinson 1992 | Devon Rex | 10-14 weeks | Exercise intolerance. Peculiar gait, high stepping. Feeding difficulties. Condition deteriorates or stabilizes. |  |  |  | Autosomal recessive inheritance pattern |
| Malik *et al.,* 1993 | Devon Rex, male | 10 weeks | Moderate muscle weakness. Stable clinical status. Died at 27 months after chocking on a large piece of meat. | Within reference ranges for routine hematology and plasma biochemistries in all four cats tested. No elevation in CK activity.  Activities of a range of mitochondrial enzymes from 2 cases and a control cat were similar. |  | Similar histological changes in all muscles examined. Extent and severity of changes varied from case to case and from muscle to muscle in a given individual. Tendency for dorsal cervical and proximal forelimb muscles to be affected more than proximal hindlimb and distal limb muscles. Histological changes were positively correlated with both the severity of signs and age at time of biopsy.  Fibres with large cross sectional areas and rounded. Small angular fibres, both singly and in small groups. Increased variation in the size of muscle fibres. Occasional degenerating fibres, with numerous histiocytes in and around them. Presence of subsarcolemmal nuclei and some fibre regeneration.  Severe dystrophic changes in muscles collected from older, more severely affected cats. High variation in fibre cross sectional areas. Increased numbers of nuclei, internal nucleation and fibre splitting. Necrotic fibres and regenerating fibres. Predominance of fast twitch (type II) fibres. No fibre type grouping. No evidence of axonal degeneration, demyelination or cellular infiltration in peripheral nerves. |  |
|  | Devon Rex, female | 11 weeks | Moderate appendicular weakness. Repeated chocking episodes during feeding. Stable clinical status. Died at 19 months after chocking. |  | Sparse fibrillation potentials and positive sharp waves in triceps brachii and dorsal cervical muscles. Normal conduction velocity of motor axons in the tibial and ulnar nerves. |  |  |
|  | Devon Rex, male | 23 weeks | Deterioration of the condition over several months. Post prandial colic, reflux oesophagitis. Respiratory infection at 18 months of age. Euthanasia  at 2 years of age. |  |  |  |  |
|  | Devon Rex, male | 6 weeks | Moderate weakness. Several chocking episodes during meals. Death at 6 months of age from laryngeal obstruction (chocking episode). |  |  |  |  |
|  | Devon Rex, male | 7 weeks | Moderate muscle weakness that worsened during winter. Several chocking episodes during feeding. Died at 20 months from upper airway obstruction. |  |  |  | Exacerbated weakness after intravenous administration  of 0.1 mg/kg of edrophonium |
|  | Devon Rex, female | 4 weeks | Near normal exercise tolerance. Slight deterioration in motor performance. |  |  |  |  |

| Martin *et al.,* 2008 | Sphynx, male | 10-16 weeks | Decreased muscle mass. Chipmunk posture. Fatigability. Died at 18 months after aspiration or chocking. | Within reference ranges for routine hematology and plasma biochemistries. No elevation in CK activity. | Spontaneous activity: fibrillation potentials and complex repetitive discharges in multiple muscle groups. Waveform configuration and motor and sensory nerve conduction velocities were within normal limits for feline peroneal nerve, and cord dorsum potentials and late waves were present. Decremental response to repetitive nerve stimulation, that was not reversed by intravenous edrophonium. | Excessive variability in myofiber size, type1 fiber predominance. Infrequent centrally located myofiber nuclei.  Unremarkable intramuscular nerve branches. No abnormalities  in nerve biopsy  sections. |  |
| --- | --- | --- | --- | --- | --- | --- | --- |
|  | Sphynx, female | 10 weeks | Required hand feeding to avoid chocking episodes. Crouching gait. Decreased muscle mass. Static disease features. Found dead at 22 months of age. | Mature neutrophilia. Decreased creatine and cholesterol. Mildly elevated CK activity  (325 UI/L; reference range 73-260 UI/L). | Fibrillation potentials, positive sharp waves and complex repetitive discharges in multiple muscle groups, and normal motor and sensory nerve conduction studies. No decremental response to repetitive nerve stimulation. | Excessive variability in myofiber size, type1 fiber predominance. Infrequent centrally located myofiber nuclei.  Unremarkable intramuscular nerve branches. No abnormalities  in nerve biopsy  sections. |  |
|  | Devon Rex, male | 6 weeks | Moderate muscular weakness. Slow progression of the disease. Loss of skeletal body mass. Lower respiratory track infections. Euthanasia at 6 years of age. | Within reference ranges for routine hematology and plasma biochemistries. No elevation in CK activity. |  | Excessive variability in myofiber  size and internal nuclei, occasional necrotic fibers with histiocytic infiltration and several regenerating fibers. Changes in the dorsal cervical muscle were more pronounced than in the triceps muscle. |  |
| This study | Sphynx, female | 3 weeks | Needed frequent resting periods when playing. No muscle atrophy. Peculiar gait while walking, with shoulder blades held high. Reduced exercise tolerance. Chocking episodes during feeding. Died accidentally at 18 months of age. | Within reference ranges for routine hematology and plasma biochemistries. No elevation in CK activity. | Weak, slightly abnormal spontaneous activity within the tibialis anterior.  Normal velocities of tibial and ulnar nerves. | Cervical muscle showed the most prominent abnormalities, including fibre size and shape variation; most of myofibres appeared round and atrophic or hypertrophic. Muscle spindles and intramuscular nerves were unremarkable. Fibres were all well differentiated. No topographic aggregation for type-1 or type-2 fibres. Slight type1 fiber predominance**.** Loss of acetylcholinesterase clustering at the neuromuscular junction. |  |
|  | Sphynx, male | 4 weeks | Severe muscular weakness. Reduced exercise tolerance. Chocking episodes. Lower respiratory track infections. Euthanasia at 2 years of age. |  |  |  |  |
|  | Devon Rex, female |  | Muscular weakness. Reduced exercise tolerance. |  |  |  |  |

CK: creatine kinase.

Common clinical features to all cases: - passive ventroflexion of the head and neck

- appendicular weakness

- reduced exercise tolerance

- dorsal elevation of the scapulae
